# PheNominal: an EHR-integrated web application for structured deep phenotyping at the point of care

**DOI:** 10.1186/s12911-022-01927-1

**Published:** 2022-07-28

**Authors:** James M. Havrilla, Anbumalar Singaravelu, Dennis M. Driscoll, Leonard Minkovsky, Ingo Helbig, Livija Medne, Kai Wang, Ian Krantz, Bimal R. Desai

**Affiliations:** 1 Center for Cellular and Molecular Therapeutics, Children’s Hospital of Philadelphia, Philadelphia, PA 19104 USA; 2 Emerging Technology and Transformation Team, Information Services, Children’s Hospital of Philadelphia, Philadelphia, PA 19104 USA; 3 Division of Neurology, Children’s Hospital of Philadelphia, Philadelphia, PA 19104 USA; 4 The Epilepsy NeuroGenetics Initiative (ENGIN), Children’s Hospital of Philadelphia, Philadelphia, USA; 5 Department of Biomedical and Health Informatics, Children’s Hospital of Philadelphia, Philadelphia, PA 19104 USA; 6 Department of Neurology, University of Pennsylvania, Perelman School of Medicine, Philadelphia, PA 19104 USA; 7 Roberts Individualized Medical Genetics Center, Children’s Hospital of Philadelphia, Philadelphia, PA 19104 USA; 8 Department of Pathology and Laboratory Medicine, University of Pennsylvania Perelman School of Medicine, Philadelphia, PA 19104 USA; 9 Department of Pediatrics, University of Pennsylvania Perelman School of Medicine, Philadelphia, PA 19104 USA

**Keywords:** EHR, EMR, Phenotype, Epic healthcare, Health record data

## Abstract

**Background:**

Clinical phenotype information greatly facilitates genetic diagnostic interpretations pipelines in disease. While post-hoc extraction using natural language processing on unstructured clinical notes continues to improve, there is a need to improve point-of-care collection of patient phenotypes. Therefore, we developed “PheNominal”, a point-of-care web application, embedded within Epic electronic health record (EHR) workflows, to permit capture of standardized phenotype data.

**Methods:**

Using bi-directional web services available within commercial EHRs, we developed a lightweight web application that allows users to rapidly browse and identify relevant terms from the Human Phenotype Ontology (HPO). Selected terms are saved discretely within the patient’s EHR, permitting reuse both in clinical notes as well as in downstream diagnostic and research pipelines.

**Results:**

In the 16 months since implementation, PheNominal was used to capture discrete phenotype data for over 1500 individuals and 11,000 HPO terms during clinic and inpatient encounters for a genetic diagnostic consultation service within a quaternary-care pediatric academic medical center. An average of 7 HPO terms were captured per patient. Compared to a manual workflow, the average time to enter terms for a patient was reduced from 15 to 5 min per patient, and there were fewer annotation errors.

**Conclusions:**

Modern EHRs support integration of external applications using application programming interfaces. We describe a practical application of these interfaces to facilitate deep phenotype capture in a discrete, structured format within a busy clinical workflow. Future versions will include a vendor-agnostic implementation using FHIR. We describe pilot efforts to integrate structured phenotyping through controlled dictionaries into diagnostic and research pipelines, reducing manual effort for phenotype documentation and reducing errors in data entry.

**Supplementary Information:**

The online version contains supplementary material available at 10.1186/s12911-022-01927-1.

## Background

Phenotypic data, especially in the electronic health records (EHR), is heterogeneous and sparse [1–3]. The data comes in numerous different formats that do not facilitate interoperability or direct comparison of information [4–7]. Having a standardized, controlled phenotype vocabulary and data format is preferable to distinguish phenotypic groups and obtain a diagnosis [8]. Distinct phenotype ontology terms have already been used in several studies [9–16] to facilitate diagnosis of rare diseases and identification of causal genes. There is an acute need in clinical settings for well-structured phenotype data so that it can be combined with downstream gene panel, SNP array, or sequencing data for rapid, accurate comprehension of both common and rare diseases [17, 18]. Some tools can even use this data to rank genes without any sequencing information [18, 19], which can direct sequencing, gene panels, or downstream analyses.

To obtain distinct phenotype terms, we use data from standardized phenotype vocabularies, but the most common and widely utilized vocabulary is the Human Phenotype Ontology (HPO) [20]. The Human Phenotype Ontology was created to enable “deep phenotyping” through the capture of symptoms and phenotypic findings using a logically constructed hierarchy of phenotypic terms and enables a deep phenotyping approach wherein computable phenotypic profiles of human diseases and individual patients allow the linking of terms that are close to one another in the hierarchy and provides for a computational bridge between genome biology and clinical medicine. HPO has become the de facto standard for representing clinical phenotype data in a multitude of programs including the NIH Undiagnosed Diseases Program (UDP) [21], several NCBI databases including MedGen [22], ClinVar [23], and the Genetic Testing Registry [24], the Sanger Institute databases DDD [25] and DECIPHER [26], the rare diseases section of the UK's 100,000 genome project [27], the Genomic Matchmaking API of the Global Alliance for Genomics and Health [28], and many others. The UDP demonstrated that the use of HPO in comparison to clinical data alone in ES/GS variant analysis improves molecular diagnosis by 10–20% [29], as has several other studies [26, 30–32]. HPO provides a substantially more detailed representation of clinical phenotypes than other clinical terminologies and ontologies and is designed for computational analysis by linking to computational disease definitions and to ontologies of gene function, anatomy, biochemistry, and other biologic attributes.

Despite the improvements provided by HPO, it can be difficult to train people in the use of standardized ontologies, and natural language processing (NLP) tools can be difficult to integrate and vary greatly in consistency [18, 33, 34]. Several extracted terms are also near-synonymous and not merged depending on the ontology used [35]. Recently, point-of-care strategies have emerged to address these facts, such as improved testing quality [36], deeper phenotyping strategies in EHR data [37, 38], and improved clinical documentation [39], but these improvements are not yet enough.

Even with these improvements at the point-of-care, these automated tools are unlikely to achieve the same result as manual annotation by expert users (such as the patients’ physicians, specialists, or genetic counselors), and while there are resources containing integrated HPO terms for use in manual annotation such as the Human Disease Gene website [40], they are not kept up-to-date individually and either miss new or contain outdated terms. For example, the epilepsy phenotypes within the HPO were recently updated in December 2020 [41] in alignment with the most current guidelines formulated by the International League Against Epilepsy in 2017. However, if a provider uses a framework based on a static version of the HPO from 2019 it may lack such recent information, or use terms that are no longer in use in the epilepsy community. This inability to implement the most recent release rapidly can create inconsistent patient phenotyping depending on the expert user’s resource of choice.

Previously, genetic counselors and physicians at the Roberts Individualized Medical Genetics Center (RIMGC) at the Children’s Hospital of Philadelphia (CHOP) used a manual process of annotating clinic encounter notes with tags from the HPO website. Providers copied and pasted HPO codes into encounter progress notes, often with errors, and while they were easy to find they were not discretely captured. We have created PheNominal to remedy this deficiency. PheNominal is a tool to assist expert users in annotation of patient notes with preexisting phenotype terms from HPO. In addition to raw patient notes, users can now extract a full set of HPO terms curated by the physicians themselves in easily parsable formats for downstream bioinformatics pipelines. In cases where genetic variant data is attained for patients, there are a variety of tools that can utilize this deeper, more normalized phenotype data to rank candidate genes for variants [19, 42–44].

With this tool, we hope to create a more consistent and standardized method for expert curation of phenotype terminology for reproducibility and dissemination to bioinformatics pipelines. In this study, we describe the tool development process and how to use the tool at point-of-care. We demonstrate how the tool has improved the accuracy, speed and willingness of physicians in phenotyping their patients at a major genetic testing center for pediatric patients. Finally, we provide a realistic example of how discrete, structured phenotyping of patients can lead to a genetic diagnosis in disease.

## Materials

### Development

Using the Agile software methodology and a series of development sprints [45], the Emerging Technology and Transformation Team actively involved customers in the product development process and continually implemented their feedback and rapidly tested each feature. The goal of the design was to create an interface that was easy to use for clinicians with no specialized technical background, particularly those less familiar with various ontological databases. The development team worked with providers at the RIMGC at CHOP to design and validate the interface. Autocompletion was added to deal with common typographic errors that led to incomplete or inaccurate annotations and increased note taking time. Physician suggestions, such as adding comments to the HPO terms for negation and gene updates, were critical. Providers were given biweekly demonstrations to assess progress, perform tests and work with the product; provider feedback was clear and concise as requirements were clearly specified for feedback, and every new feature was tested immediately in production by physicians and counselors.

### Typical use case of PheNominal

**Fig. 1:**
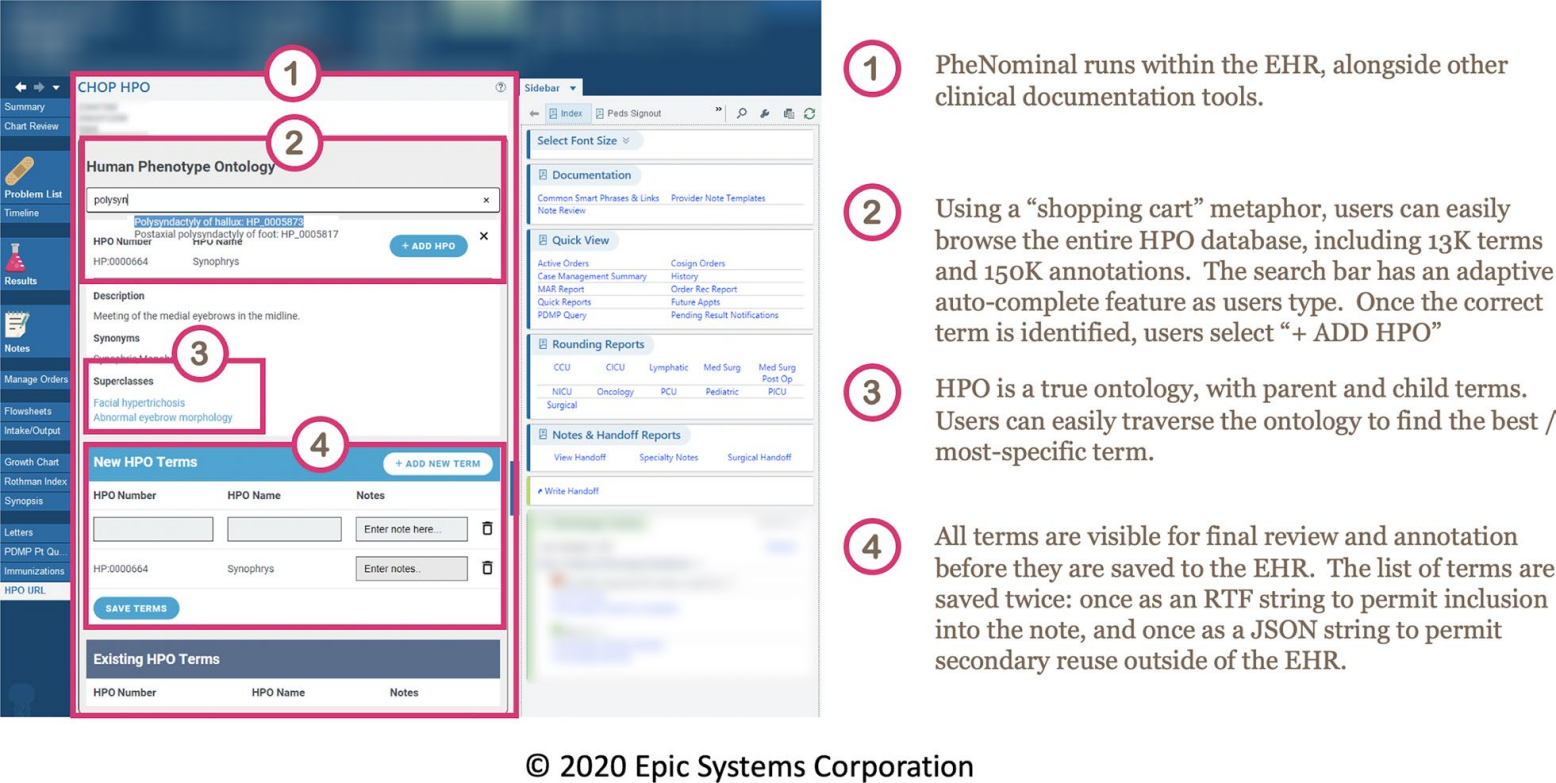
PheNominal basic use case. In part 2, the user is considering the addition of 2 discrete HPO terms, synophrys (unibrow) and polysyndactyly of hallux (extra big toe) for their patient. In part 3, the superclass and subclass terms are highlighted, as in some cases the user may prefer to be more or less specific in their term choice. After adding terms in part 2, in part 4 the user may choose to save the terms to the EHR, which makes them immediately available for inclusion in patient notes as a pre-formatted Rich Text Format (RTF) table, as well as available for downstream use in bioinformatics or analytics pipelines

The PheNominal app is smoothly integrated into the Epic electronic health record (Epic Systems Corporation, Verona, WI), and currently available as a tab labeled “HPO URL” in the Epic Hyperspace once the patient chart is opened. The application works akin to a shopping cart for an online website (Fig. 1). Training in using the tool involves a brief live demo for prospective users on how they can search and enter a HPO term for the patient and some troubleshooting guidelines. Users have access to the entire HPO vocabulary, which is kept up-to-date: as of January 2021 it has over 13,000 terms and 156,000 annotations. The user can browse the full scope of HPO simply by typing the initial letters of a term, using the “autocomplete” feature as an accelerator, selecting the term, and clicking “Add HPO” to confirm selection. Users can browser the HPO hierarchy to find the correct level of precision by clicking “Details,” which reveals the related synonymous, superclass, and subclass terms, as well as the description of the term and gene annotation data for the term from Entrez [46] and HGNC [47] (Fig. 1, Part 3).

**Fig. 2:**
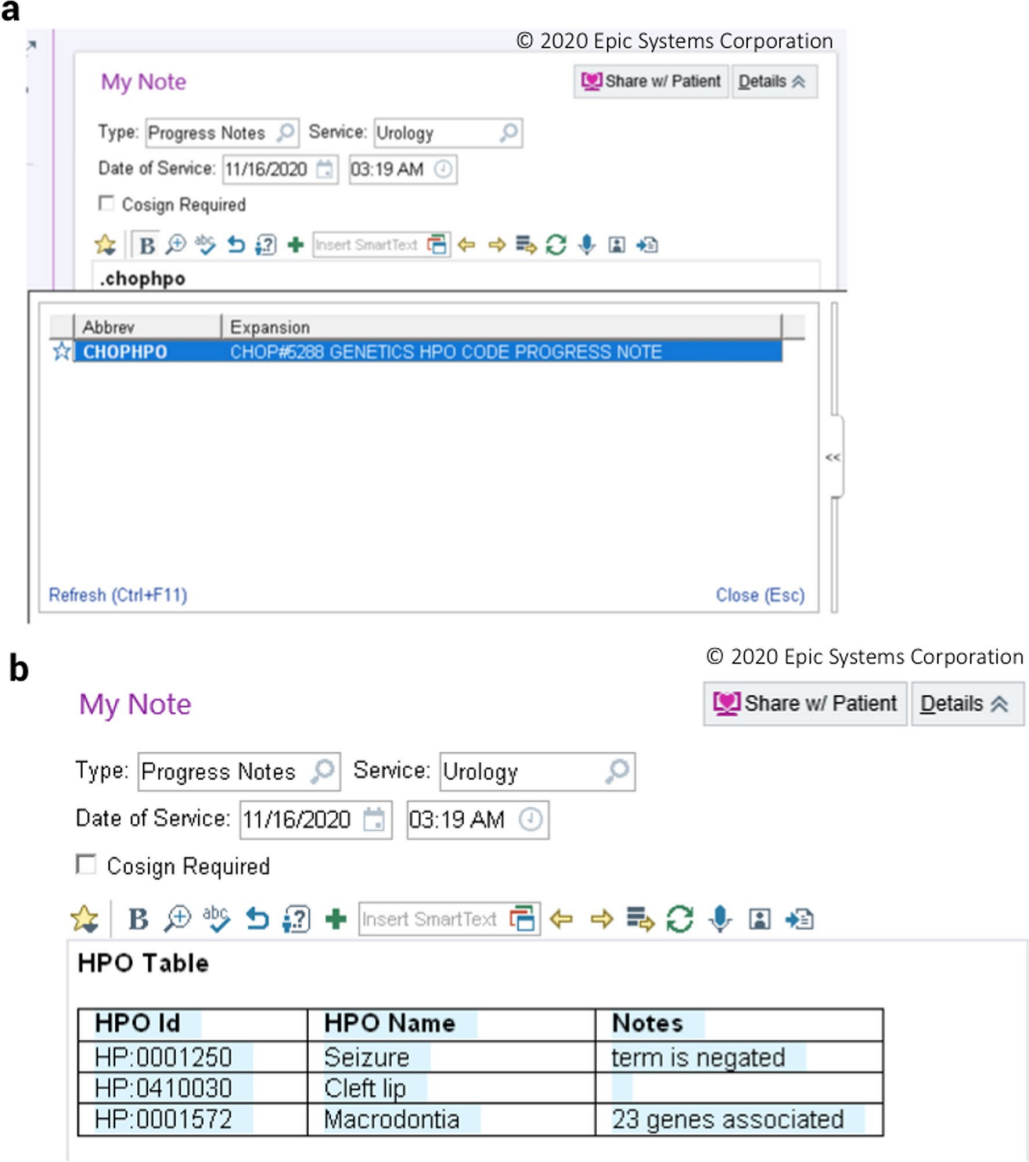
Integrating HPO terms from PheNominal into patient notes as RTF tables. **a** HPO terms are stored as two discrete SDEs: one, a JSON-formatted payload containing date and version information, and the other, **b** a simplified RTF table intended for use in clinical notes. Including the SmartLink “.chophpo” in a clinical note pulls the preformatted RTF table with annotations into the note itself

As the user adds terms to the “shopping cart,” they can add free-text annotations to each term, such as linked genes, or if the term is a negated term (Fig. 1, Part 4). Clicking “Save Terms” saves the information to “Existing HPO Terms.” The full set of HPO terms and annotations are formatted as a single JavaScript Object Notation (JSON) string, which is then stored in the Epic Chronicles database as an Epic SmartData Element (SDE). An SDE is a vendor-specific technique for storing discrete key-value pairs in Epic such that important phenotype information can be stored consistently, reproducibly, and discretely. New, modified, or removed terms are saved to the patient's medical record after updating terms as well (Additional File 1, demo at: PheNominal Demonstration and Tutorial). Because the Epic EHR has standard web service methods to access and write SDEs as well as methods to manipulate SDE data within a clinical note using “SmartLinks,” we are able to format and present an RTF formatted tabular view of the JSON data for use in Epic notes, retrieve discrete HPO terms for use in downstream bioinformatics and pipelines, and query patient records for specific terms in the enterprise data warehouse (Fig. 2, Additional file 1).

### App architecture and design

The app was developed in JavaScript and CSS using Node.js. It communicates in real-time with Epic Caché as well as the Bioontology API from BioPortal, which gives us the benefit of access to the very latest version of the HPO ontology and its terms. We also maintain a local version of the HPO ontology for performance that is also kept up-to-date via mailing lists and automated server queries. Users can benefit from access to fully updated terms as well as outdated terms if necessary, as all saved terms are version-tagged and permanently stored in the electronic health record unless updated by an authorized user.

**Fig. 3:**
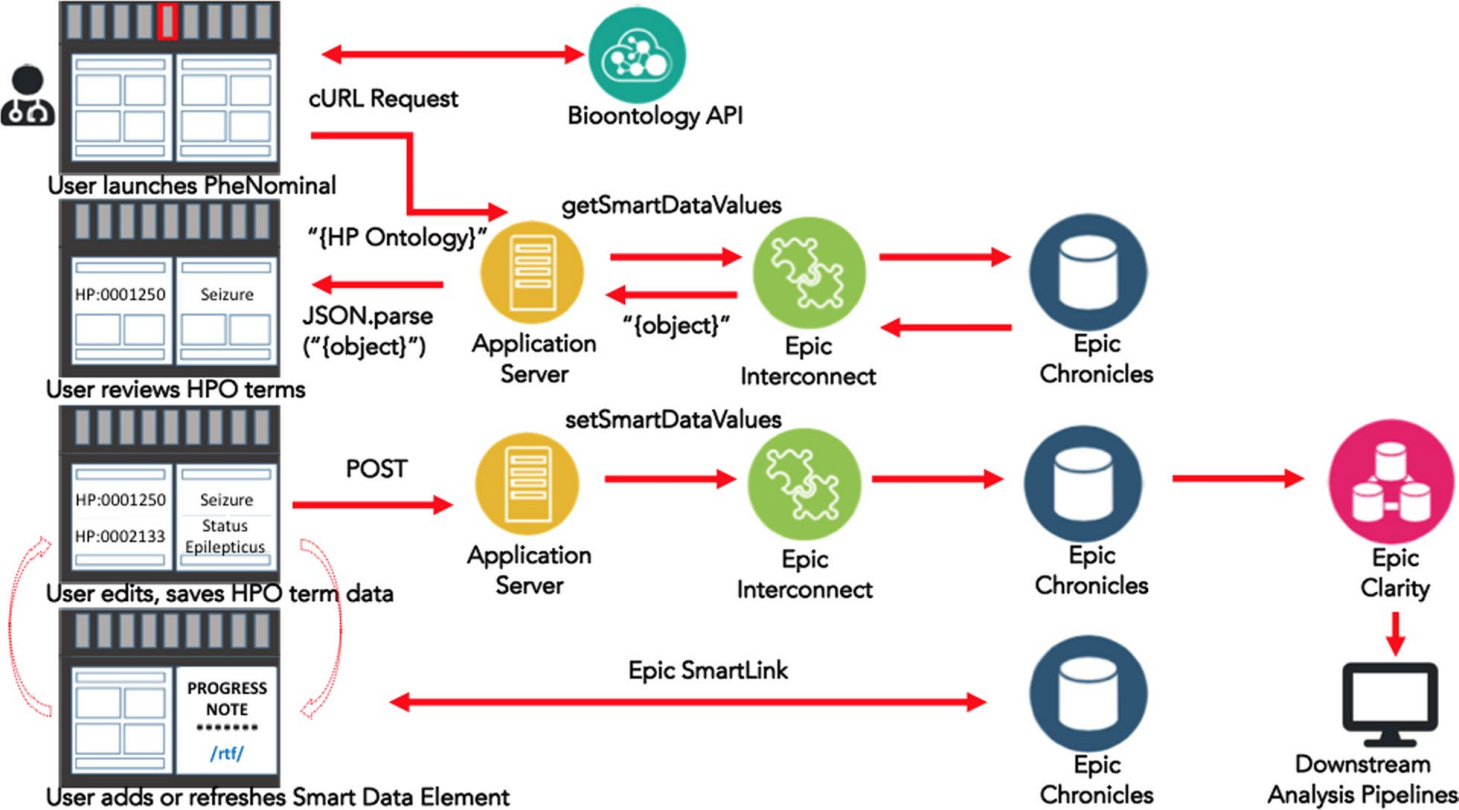
PheNominal system architecture and workflow. Users can search, review, add and delete HPO terms by having the Progress Notes open side by side for clinical analysis. The search is based on an external API built by NCBO BioPortal, which gets the HPO data from the HPO site. We store a copy of the full HPO data locally into our local application server and use it to populate the HPO details such as HPO name, description, synonyms, superclasses, subclasses and HPO gene association. Terms are discretely stored in the EHR, and pushed to Epic Chronicles, and daily to Epic Clarity and our proprietary CHOP data warehouse, and can be imported into the clinical note directly for use in other downstream pipelines, or downloaded from Clarity by direct SQL query by authorized researchers

PheNominal uses two vendor-specific web services (getSmartDataValues and setSmartDataValues) to access and write HPO terms as SDEs (Fig. 3). When the end user launches PheNominal, the tool retrieves and parses the most current HPO term set from NCBO BioPortal via the Bioontology API [48]. As the user manipulates HPO terms in the interface, changes are relayed to the application server to send and receive relevant data. The web services communicate via Epic Interconnect to generate two SDEs, a JSON payload and a pre-formatted RTF table for inclusion in clinical notes. If the user decides to save the term, this is updated downstream by adding the SDE to our Epic Clarity database for the patient. Finally, as in Fig. 2, Epic Smartlink allows for easy insertion of SDEs into patient notes.

## Results

We collaborated with the Emerging Technology and Transformation team of the IT department and the clinicians and counselors of the RIMGC at CHOP to assess the improvement of PheNominal over manual entry of phenotype terms in number, accuracy, and speed. The IT team members were only the designers of the tool, and the RIMGC clinicians and counselors were the users. Before PheNominal, RIMGC clinicians had to navigate to the HPO web page manually, copy HPO terms and IDs, return to the Epic Hyperspace to paste them into patient notes without correcting them, or even type them out manually. This had an unacceptably high error rate for reliable inclusion in bioinformatics pipelines. During an effort to manually convert free text HPO terms from manually entered, historical notes into discrete HPO terms, the development team found thousands of HPO terms that had incorrect annotations, missing data, or typographic errors. Forty of these records were so corrupted they could not be migrated programmatically. The need for a tool like PheNominal became abundantly clear early on in the migration process.

In over almost 5 years of the legacy system of manual SmartPhrase input, only 1175 individuals’ records were annotated with HPO terms. But in the 1 year and 4 months since PheNominal’s implementation in the system, over 1500 patients’ records were annotated (Table 1). When the Legacy system was in place, HPO terms were only assigned to patients undergoing exome sequencing tests. Since the implementation of PheNominal, HPO terms have been assigned to all patients undergoing any clinical evaluation as well as those undergoing any genetic testing through the RIMGC program. The ease of use of the PheNominal app allowed it to be applied to the entire patient population evaluated by the RIMGC clinic, which is the primary reason for the notable increase in the number of patients served. In a little over a year after PheNominal’s implementation, 1000 more HPO terms were saved into Epic accurately than in nearly 5 years of manual input, with 3 times the average speed at 5 min per patient. The other benefit of having an automated app with autocompletion is it ensures all terms are entered correctly, and thus discretely, for future pipelines and downstream analyses. In addition all terms must be entered and viewed on an encounter-by-encounter basis for each patient in the legacy system, but with PheNominal, all HPO terms can be edited and viewed simultaneously for convenience and speed. It is important to note that there was no difference between the patient populations served by the Legacy system and PheNominal, nor in the clinical teams doing the HPO annotation: for each case the annotation was performed by a genetic counselor and reviewed by a physician geneticist.

**Table 1:** Comparison between legacy system (manual entry of SmartPhrases) and PheNominal on patient encounters. This compares the total measurement period for each system, number of patients served, the number of HPO terms entered correctly, how long each method takes on average, and how terms are viewed and if they are truly discrete in all cases

	Legacy (manual entry)	PheNominal
Time	4 years, 9.5 months (09/01/2014–06/19/2019)	1 year, 7.5 months (06/19/2019–1/31/2021)
Patients served	1175	1760
HPO terms saved	10,050	13,566
Time to enter terms	15 min mean	5 min mean, 2 min mode
Viewing terms for a patient	Each encounter must be opened individually	All at once across encounters
Discrete?	No	Yes

**Fig. 4:**
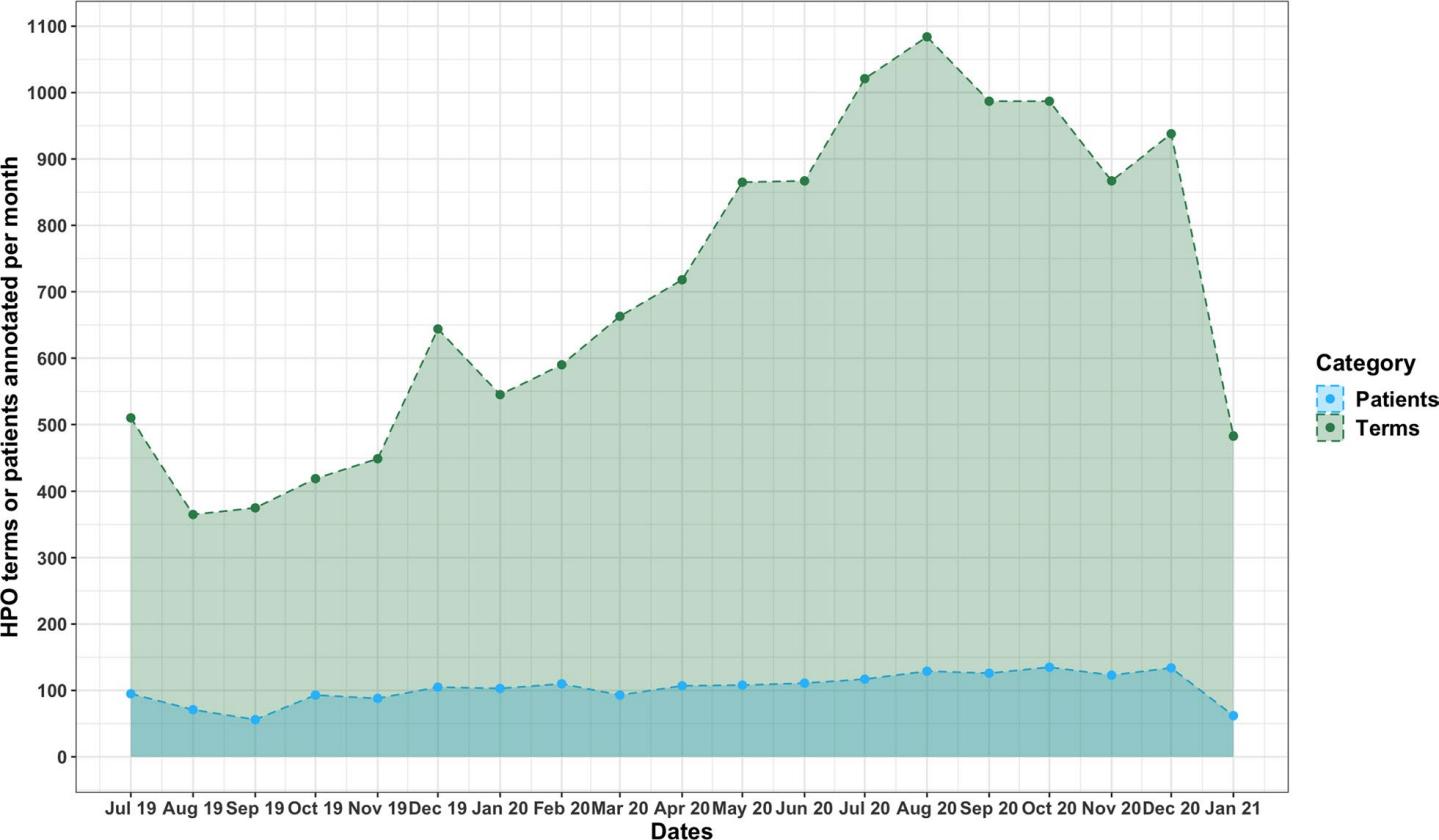
Distribution of HPO terms, and patients served at the Roberts Individualized Medical Genetics Center at the Children’s Hospital of Philadelphia after the initial implementation of PheNominal implementation from July 2019 to January 2021. The green line number of unique patients with HPO terms annotated by PheNominal per month. The blue line is the number of HPO terms annotated for all patients per month by PheNominal. This figure was generated in R using the abovementioned data gathered from the RIMGC directly

After PheNominal was implemented around the end of June 2019, the number of patient records annotated per month only went up slightly over time, but the HPO terms annotated per patient substantially increased (Fig. 4). This is partially due to recruitment of more patients outside of simple clinical testing over time but primarily because there was a marked increase in comfort by physicians and counselors using the tool to find the most specific and descriptive terms as the users familiarized themselves with the ontology. We believe, while difficult to quantify, that this factor contributed strongly to the large gradual increase in HPO term count per month over time, which is not explained by the far less appreciable increase in patient count per month.

By virtue of PheNominal, not only are there less errors due to the discrete choices provided by the app, but now users can traverse parent and child HPO terms in the ontology tree with ease. For example, as in the HPO paper from Robinson et al. [49], a physician can navigate the tree and where they would previously annotate “hip dislocation” they can now write “congenital bilateral hip dislocation.” Alternatively, a user may wish to be less specific, when they do not have enough facts to identify a specific feature. PheNominal therefore makes overall diagnosis easier, and assists in accurate gene-phenotype association for downstream bioinformatics and sequencing pipelines.

**Fig. 5:**
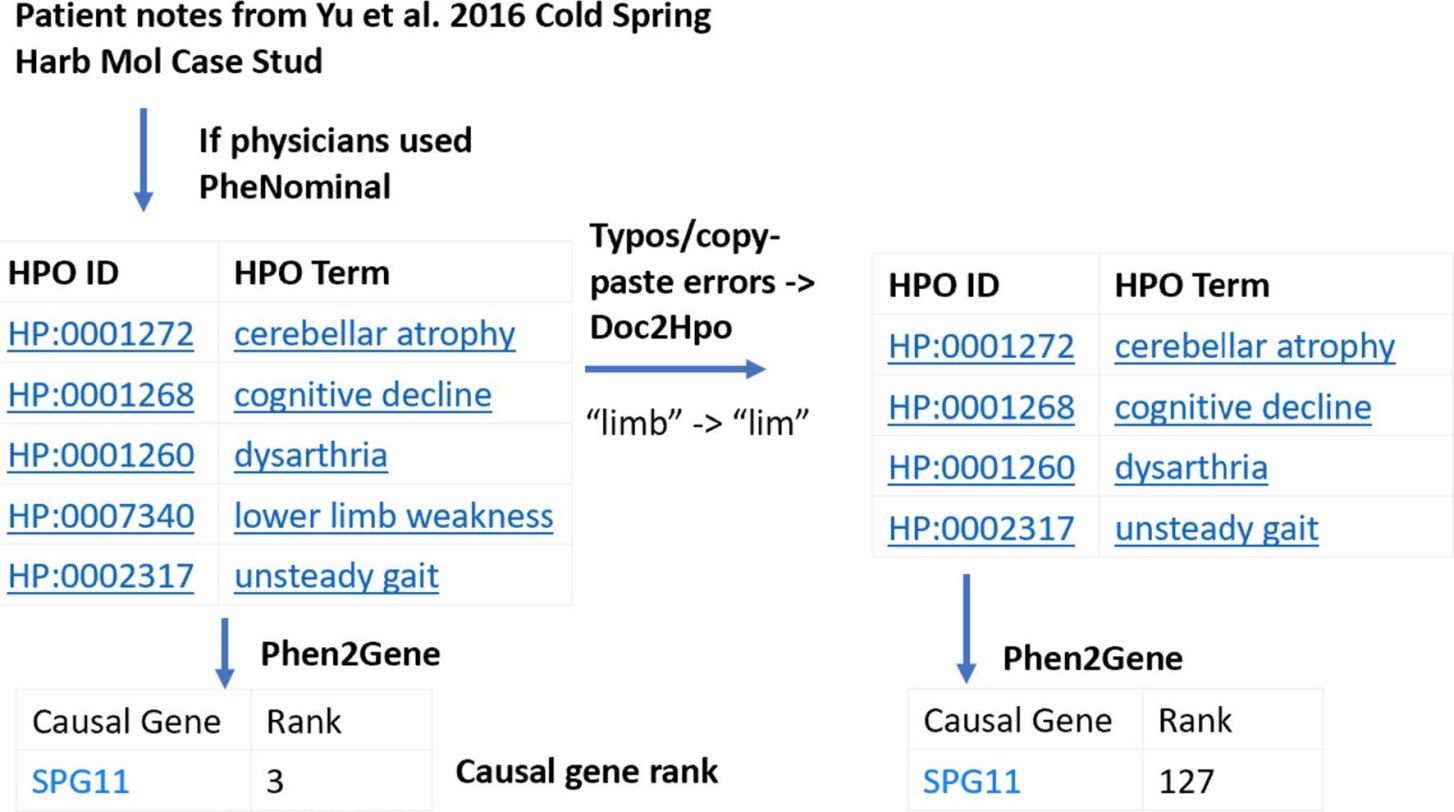
Inputting terms with the support of PheNominal versus manual extraction downstream with NLP tools. In this example, we derived several HPO terms derived from patient notes with the help of PheNominal. We provide realistic examples of 3 reproducible copy-paste errors like “limb” to “lim” that produce missing terms when parsed by NLP algorithms such as Doc2Hpo. The derived HPO terms are then passed to Phen2Gene, which ranks potential disease genes based on phenotype information alone. In this example, the rank drops from 3 to 127, which may result in a variant in a candidate gene (*SPG11*) not to appear on the final diagnostic report

As an example of how PheNominal assists in downstream analysis, we took a sample of unannotated patient notes from Yu et al. on a patient with hereditary spastic paraplegia [50] (Fig. 5). We can add the terms from the patient notes with PheNominal, under the assumption it would prevent input error with its autocompletion, which is the set of HPO terms seen on the left side of Fig. 5. However, if we allowed physicians to type the notes or manually enter or copy-paste the HPO terms into patient notes and parse them using the basic Aho-Corasick algorithm from Doc2Hpo, we could run into some preventable user errors, such as pasting incomplete terms or misspelling terms. As mentioned previously, the RIMGC produced thousands of incorrect annotations. For the sake of argument, if these mistakes remove even 1 term from the 5 term list in Fig. 5, we go from scoring the causal gene (without any genetic variant knowledge) in the top 3 genes, to scoring it in the top 127 genes, using Phen2Gene in our downstream pipeline. PheNominal is critical for preventing simple errors like these that can seriously hinder or prevent the proper diagnosis of disease for troubled patients.

## Discussion

Here, we report PheNominal, a point-of-care web application, embedded within Epic electronic health record (EHR) workflows, to permit capture of standardized phenotype data. PheNominal has improved the speed and ease with which physicians and genetic counselors can input discrete, reliable phenotypic data in the EHR. Specificity of terms can now be easily modulated, and the more specific an HPO term, the more information it contains. There are clear advantages of using PheNominal to consistently and reproducibly capture discrete HPO terms for downstream use in computational pipelines, as opposed to the makeshift post-hoc extraction of the past. Thanks to PheNominal, annotation mistakes and sparse phenotyping made by manual copy-and-paste entry can become an ancient memory for all healthcare systems and providers.

Future improvements to PheNominal will address limitations in compatibility, interoperability and comprehensiveness of the tool. Because PheNominal acts as a general-purpose ontology browser and annotation tool, it can be expanded to include other ontologies beyond HPO. We can also provide sorted autocomplete information that is ranked by relevance so that users are less likely to choose the wrong term, so we are not simply substituting typing errors for term selection errors. Term recommendations may be further improved by future work in NLP tools that can predict potential HPO terms based on a set of already chosen terms, though at the moment no tools can fulfill this purpose. Additionally, while PheNominal currently uses vendor-specific web services to read/write Epic SDEs, there is strong motivation to port the platform to use FHIR resources to improve cross-platform interoperability.

Because EHR vendors differ in their implementation of FHIR, and because PheNominal is dependent on the use of SDEs to store raw JSON data, there are some important considerations for future FHIR integration efforts. Some of the point-of-care affordances, such as the real-time availability of the entered terms in clinical notes, are harder to port to other EHRs or in a pure FHIR app, and the SmartPhrase concept may no longer be supported in all cases, which makes porting currently annotated Progress Notes difficult. But while the FHIR standard is still going through major changes, early conversations with the FHIR Genomics Working Group at CHOP have been positive and suggest a few potential options for conversion to FHIR-based resources.

Integrating new information into PheNominal is relatively easy. There is already an API in place for Phen2Gene [19] to take the gene annotations from PheNominal and return scores for each term within a second. With Phen2Gene ranking genes, we can sort by score, and prioritize potentially causal genes for physicians and genetic counselors at the point of care. Since we are using the Bioontology API, porting other ontologies contained in NCBO BioPortal is also easy: OMIM [51], SNOMED [52], MeSH [53], ICD-10 [54], ORDO [55], and DOID [56]. It is also entirely possible to combine other data collection tools at the point of care with the help of PheNominal and with the help of SDEs, integrating this information into downstream clinical workflows for Clinical Decision Support (CDS). We hope that clinicians and counselors will find this application a useful resource for improving the sensitivity and reproducibility of phenotypic annotation, and would use it to improve diagnosis speed and accuracy at the point of care.

## Conclusion

PheNominal is a pilot effort to incorporate structured phenotype information using precise dictionaries into downstream diagnostic and research pipelines by reducing manual input during phenotype documentation and generation of patient notes. This reduces errors in data entry that lead to more accurate downstream results in delineating the subphenotype, and predicting candidate genes for disease.

We believe there are 5 main innovations of PheNominal. It is a tool for discrete, point-of-care capture of clinically precise phenotype terms with minimal effort. It utilizes secure and standard-compliant encoding and storage of the HPO terms directly into the EHR. The data is dual-formatted both to permit downstream reuse in JSON format, containing date and version information, as well as to allow point-of-care insertion into a clinical note in RTF format. PheNominal is integrated into the EHR directly through native web services, permitting generalizability to any other EHR implementations and the FHIR standard. Lastly, it permits integration of other ontologies and evolving standards like Phenopackets.

## Supplementary Information

**Additional file 1.** PheNominal Demonstration and Tutorial.

## Data Availability

There are no new data associated with this article, as no new data were generated or analyzed in support of this research. Patients annotated with the tool cannot have their annotation data shared for HIPAA privacy reasons. The software tool was developed at The Children’s Hospital of Philadelphia, and is available with appropriate institutional usage and license agreement.

## Abbreviations

EHR: Electronic health record
FHIR: Fast healthcare interoperability resources
HPO: Human phenotype ontology
CDS: Clinical decision support
RTF: Rich text format
API: Application programming interface
SDE: SmartData Element
CHOP: Children’s Hospital of Philadelphia
